# Put a cork in it: using Instillagel to prevent urine spillage during suprapubic catheter insertion

**DOI:** 10.1308/rcsann.2025.0044

**Published:** 2026-01-20

**Authors:** R Karanjia, A Chetwood, D Whiting

**Affiliations:** NHS Frimley Health Foundation Trust, UK

## Background

Suprapubic catheter (SPC) insertion requires a distended bladder for safe insertion and to avoid the risk of bowel injury. This is performed either as an emergency, often at the bedside, or in theatre where the bladder is distended artificially during cystoscopy. A Seldinger technique is now widely adopted due to its safety. However, there is often a scramble to insert the catheter after the puncture to prevent large volumes of urine from the distended bladder spilling all over the patient and the bedside.

## Technique

After the puncture has been made, the puncturing component of the SPC is removed, leaving an open channel to the bladder. Instead of attempting to quickly insert the catheter, a tube of Instillagel is inserted into the channel upon removal (Video 1). The viscosity of the gel prevents urine from spilling everywhere for up to 20 seconds, even under high pressure, and a catheter can be inserted calmly without risk of spillage. The technique usually yields high praise from the nursing team, who now do not have to clear up the expected mess or change the patient's wet clothes.

## Supplementary information

The online version contains supplementary material available at https://doi.org/10.1308/rcsann.2025.0044.

